# Perceived facilitators and barriers to routine utilisation of standardised outcome measures among physiotherapists in Namibia

**DOI:** 10.4314/gmj.v59i4.9

**Published:** 2025-12

**Authors:** Matthew Chiwaridzo, Farirai Kamba, Zenra Buys, Munyaradzi Chimara, Marius van der Merwe, Witness Mudzi

**Affiliations:** 1 University of Namibia, Faculty of Health Sciences and Veterinary Medicine, School of Allied Health Sciences, Department of Occupational Therapy and Physiotherapy, Windhoek, Namibia; 2 University of Namibia, School of Allied Health Sciences, Department of Physical and Sport Sciences, Windhoek, Namibia; 3 University of Free State, Centre for Graduate Support, Bloemfontein, South Africa

**Keywords:** Physiotherapy, Namibia, Barriers, Facilitators, Outcome measures

## Abstract

**Objective:**

To determine the context-specific perceived facilitators and barriers to routine utilisation of standardised outcome measures (SOMs) by physiotherapists (PTs) in Namibia.

**Design:**

Questionnaire-based cross-sectional survey.

**Setting:**

Private or public clinics/hospitals in Namibia

**Participants:**

Practising physiotherapists

**Main outcome measures:**

The study evaluated “routine” utilisation of SOMs operationally defined as using SOMs for 70% to 100% of the time in clinical practice. The study also determined the perceived facilitators and barriers to the routine utilisation.

**Results:**

Of the 99 respondents, 96 (96.9%) had complete questionnaires. The majority of participants were female (n=64, 66.7%), had a Bachelor's degree (n=86, 89.6%), trained outside Namibia (n=82, 85.4%), and were practising as private practitioners (n=74, 77.1%). About half of the participants (n=47, 49.0%) “routinely” used SOMs, especially impairment-based tools. The most common reasons for SOMs utilisation were tool affordability and availability. However, about a quarter (n=41, 43.6%) were strongly deterred from using SOMs because of the language of construction of the tool, whilst 36.2% (n=34) highlighted that most SOMs had a higher reading proficiency than the patient population. Time-related barriers were also perceived by 34.0% (n=32) of the participants.

**Conclusions:**

Routine utilisation of SOMs by PTs is below average in Namibia, primarily due to tool-related factors, such as the language of construction.

**Funding:**

None declared

## Introduction

Worldwide, regulatory authorities recommend that physiotherapists (PTs) use outcome measures all the time to evaluate the responsiveness of the patients' baseline condition to the instituted physiotherapy interventions.^1^ Critical appraisal of data obtained from standardised outcome measures (SOMs) has influenced national health policies by providing information important for improving patient care approaches, supporting critical decision making and enforcing quality assurance systems.^2^,^3^ This importance creates a continued need for studies exploring utilisation rates, perceived facilitators or barriers to routine utilisation by PTs, especially in countries such as Namibia, where physiotherapy is still expanding.

Globally, PTs use the International Classification of Functioning, Disability and Health (ICF) as a conceptual framework for patient assessment.^4^ This framework allows for assessment of key health outcomes related to patients' impairments, activity limitations, participation restrictions and contextual factors affecting function.^5^ Therefore, the various types of SOMs utilised in clinical practice should reflect all the ICF domains, however, with particular emphasis on function as highlighted in the World Physiotherapy (WP) scope of practice.^6^ PTs are known to restore, develop and maintain function and optimise patients' participation in life activities.^4^ Moreover, WP emphasises evaluating changes in patients' health-related quality of life (HRQoL) as a key health outcome in physiotherapy.^6^ Currently, it is unknown whether PTs in Namibia routinely utilise SOMs, if so, which type, and to what extent the SOMs reflect ICF domains or the WP scope of practice. Without documented evidence on the contextual utilisation of SOMs and possible factors influencing usage, no policy changes can be instituted. With time, the profession potentially becomes vulnerable to stagnancy, especially in this era of increased emphasis on accountability, patient-centred approach and evidence-based practice.^2^ Therefore, research investigating the utilisation rates of SOMs, perceived facilitators and barriers in Namibia potentially provides evidence needed to guide policy formulation and apply necessary improvements.

Numerous studies investigated perceived facilitators and barriers towards utilisation of SOMs by PTs, providing a global perspective to the problem.^3^, ^7^-^11^ In Africa, however, such evidence is limited and has emanated largely from countries such as Egypt, Ghana, Nigeria and South Africa where PT services are guided by established practice guidelines, equally recognised within the public or private sectors and have been in existence for long.^7^,^9^,^10^,^12^ Comparatively, the Namibian situation is different in that there are no established practice guidelines developed or adopted for implementation by local regulatory authorities clearly mandating PTs to use SOMs. Moreover, physiotherapy in Namibia is vibrant in the private sector compared to the public.

This is partly due to a lack of registered PTs manning public PT departments. At the time of data collection, there were only four (4) registered PTs between the two public hospitals in the Capital City, Windhoek. This staff situation is probably similar to or worse than that of other regional public hospitals in the country and potentially influences utilisation rates when compared to other countries. The literature supports that the utilisation of SOMs is context-specific; hence, direct extrapolation of perceived facilitators or barriers observed elsewhere to represent factors promoting or hindering SOMs utilisation in another setting is impractical.^13^ This warrants the conduction of such studies in countries such as Namibia, characterised by a contrasting landscape to other countries with regard to PT development.^14^

The global literature reports varied utilisation rates of SOMs among clinical PTs, largely depending on study population characteristics and country.^3^,^13^ However, low to moderate utilisation rates have been reported, with various factors highlighted affecting usage. 3,7,10,15 For example, a study conducted in Ghana reported a utilisation rate of 47.6%.^9^

n that study, PTs cited SOMs' unavailability, time constraints, and increased workload as factors hindering utilisation. In another multi-national study, a 27% “all-time” utilisation rate among 241 PTs sampled from 13 French-speaking countries in Sub-Saharan Africa was reported.^15^ The main barriers highlighted in that study were lack of time, administrative support and culturally sensitive tools. All these hindrances were also reported in systematic reviews and other primary research studies. 2,3,7,13,16 With utilisation rates contextually dependent, studies aimed at understanding the perceived facilitators and barriers towards routine utilisation of SOMs by PTs are necessary, especially in countries where physiotherapy services are still expanding. In Namibia, little is known about utilisation rates, commonly used SOMs, the ICF domains they represent, and the reasons that facilitate or hinder their use. Therefore, this study sought to identify context-specific facilitators and barriers to the routine utilisation of SOMs by clinical PTs in Namibia.

## Methods

### Research approach and target population

This study formed part of a large nationwide study conducted online between June and August 2023. A detailed description of the methodology has been presented elsewhere.^17^ Briefly, the study adopted a cross-sectional research approach targeting PTs in Namibia, including interns. Interns are graduate PTs working under the supervision of senior PTs in accredited private clinics or hospitals, but are yet to register with the local regulatory council as independent practitioners. Interns were included because they represent a future cohort of registered PTs. Therefore, understanding their current practices regarding SOMs utilisation influences the future trajectory.

### Research setting and eligibility criteria

The PTs were drawn from private or public hospitals/clinics. In Namibia, PTs practice under the Allied Health Professional Council of Namibia (AHPCNA). At the time of proposal development, 253 participants were potentially eligible. To calculate sample size, parameters such as the expected utilisation rate of 47.6%, 10% precision effect, design effect of 1, 95% confidence interval and an arbitrary non-participation rate of 30% were considered.^9^ The study required 99 participants. However, the inclusion criteria considered all participants in active practice for at least six months prior to the study.

### Instrument development

A study questionnaire was developed based on previous validated instruments.^3^,^9^,^18^ The first section enquired about the socio-demographic and work-related variables as shown in Table 1. The second section had questions on educational/professional background training in SOMs, frequency of SOMs utilisation, types of SOMs commonly used and the category of patients the tool is applied for, PTs reasons for using SOMs, and the perceived barriers. Prior to use, the tool was assessed for content/logical validity and test-retest reliability using established methodological procedures described in previous studies.^19^-^22^ The exact details of the validation protocol and the obtained psychometric properties of the instrument have been presented elsewhere.^17^

**Table 1:** Demographic and work-related information of the respondents (N=96)

Variable	N (%)
**Age category (years)**	
**20-29**	45 (46.9)
**30-39**	41 (42.7)
**40-49**	10 (10.4)
**Gender**	
**Females**	64 (66.7)
**Males**	32 (33.3)
**Educational qualification**	
**Master's degree**	10 (10.4)
**Bachelor's degree**	86 (89.6)
**Undergraduate studies^+^**	
**Studied in Namibia**	14 (14.6)
**Studied outside Namibia**	82 (85.4)
**Continuing professional education^++^**	
**Yes**	68 (70.8)
**No**	28 (29.2)
**Clinical speciality**	
Yes*	47 (49.0)
**No**	49 (51.0)
**Clinical experience (years)**	
**0-5**	49 (51.0)
**>5**	47 (49.0)
**Location of work facility**	
**Government/State Hospitals**	22 (22.9)
**Private Practice**	74 (77.1)
**Country Region**	
**Khomas**	31 (32.3)
**Other	65 (67.7)
**Age of patients predominantly seen (years)**	
**Adults (≥18)**	65 (67.7)
**Children (<18)**	1 (1.00)
**Both**	30 (31.3)
**Clinical hours of work per week**	
**1-29 hours**	33 (34.4)
**30-39 hours**	38 (39.6)
**40+ hours**	25 (26.0)
**Number of physiotherapy treatment sessions usually completed in 1day**	
**1-9 sessions**	67 (69.8)
**≥10 sessions**	29 (30.2)

* refers to having a clinical specialisation in any one of the following fields cardio-pulmonary physiotherapy, general neurology, general paediatrics, musculoskeletal, and neuro-musculoskeletal

** refers to other regions in Namibia such as Zambezi, Erongo, Karas, Kavango East, Kavango West, Ohangwena, Omaheke, Oshana, Oshikoto, Otjozondjupa, Kunene, Hardap, Omusati

++ refers to the any certificate awarded after attending a continuing professional educational courses

+ refers to whether the respondents studied in Namibia or outside for their undergraduate physiotherapy studies; Area of specialisation was based on their perceived opinion looking collectively at their professional educational training, years of clinical experience, group of patients commonly treated in daily clinical practice and the continued professional education certificates awarded to them

### Procedure

The UNAM Decentralised Ethics Committee (DEC) for the School of Allied Health Sciences gave ethical clearance (Ref #: SAH04/23). The Ministry of Health and Social Services in Namibia approved the conduct of the study. Thereafter, the list of participants and contact information was obtained from APHCNA, and an independent research assistant was assigned to email all potential participants inviting them to participate. Each participant read the information letter explaining the nature of the study and indicate willingness to participate. Reminder emails were periodically sent to all participants for the entire study duration. Data was collected from June to August, 2023 until the maximum sample size was reached.

### Statistical analysis

The Statistical Package for the Social Sciences (SPSS) version 29.0 was used for main study analysis. The screening question for SOMs utilisation was dichotomised into “*routine utilisation*” and “*not routinely utilising SOMs*”. Routine utilisation meant using SOMs 70% to 100% of the time in the last six months based on question: *“Did you ever use standardised outcome measures (SOMs) during patient evaluation, assessment or re-evalation?”* with the following responses (i) *Yes, every time (100% of the time);* (ii) *Yes, usually (about 90% of the time);* (iii) *Yes, frequently (about 70% of the time);* (iv) *Yes, sometimes (about 50% of the time)* (v) *Yes, occasionally (about 30% of the time)* (vi) *Yes, rarely (less than 10% of the time)* and (vii) *Never*. Responses (i), (ii) and (iii), qualified for the combined category of “routine utilisation”. Descriptive frequency statistics were computed for all the categorical data. The reasons for routinely utilising SOMs were further analysed thematically to generate broad categories of reasons facilitating usage.

## Results

### Profile of participants and SOMs utilisation

Of the 230 participants invited, 99 completed the online questionnaire on time. However, 96 were fully completed and used for statistical analysis. Table 1 summarises the participants' demographic and work-related variables. Further details on the sample characteristics are presented elsewhere.^17^

Briefly, the majority of PTs sampled were females, had an undergraduate degree from outside Namibia and were practising as private practitioners at the time of data collection. Regarding clinical information, most PTs reported working 30-39 hours per week, completing 1-9 physiotherapy treatment sessions per day, and predominantly treating adult patients.

Table 2 profiles the utilisation of SOMs by frequency, type of SOMs commonly used and further depicts reasons facilitating SOMs usage among PTs. All the participants indicated having had an educational or professional training in the use of SOMs. Of the 96 participants, 94 (97.9%) reported having used at least one SOM in the last six months prior to the study commencement. The frequency of utilisation of SOMs in clinical practice varied greatly among the respondents during the study period. Approximately half of the participants (49.0%, n=47) “routinely” used SOMs, with 4.17% (n=4), 15.6% (n=15) and 29.2% (n=28) of the participants using SOMs “every time”, “usually” and “frequently”, respectively. For all the participant and work-related variables shown in Table 1, the Chi-square test of association only showed a statistically significant association between routine utilisation of SOMs with gender (p=0.01) and clinical specialty (p=0.004). These results have been discussed extensively elsewhere.^17^

**Table 2:** Profile of utilisation of standardised outcome measures by physiotherapists

Questionnaire variable	Frequency, n (%)
**Utilisation of SOMs in the last six months (n=96)**	
**Never**	2 (2.08)
**Yes, every time (100% of the time)**	4 (4.17)
**Yes, usually (about 90% of the time)**	15 (15.6)
**Yes, frequently (about 70% of the time)**	28 (29.2)
**Yes, sometimes (about 50% of the time)**	24 (25.0)
**Yes, occasional (about 30% of the time)**	20 (20.8)
**Yes, rarely (less than 10% of the time)**	3 (3.13)
	
**Type of SOMs routinely used (n=47)**	
**Paper-based outcome measures (e.g. Shoulder Pain and Disability Index)**	39 (83.0)
**Performance-based outcome measures (e.g. Six Minute Walk Test)**	32 (68.1)
**Instrument-based outcome measures (e.g. Goniometry, Dynamometry)**	28 (59.6)
**Clinically-administered Tests (e.g. Manual Muscle Testing/Oxford Scale)**	47(100)
	
**Classification of SOMs by domain (n=133)**	
**Impairment-based outcome measures**	81 (60.9)
**Function or participation-oriented out-comes**	50 (37.6)
**Health-Related Quality of Life outcome measures**	1 (0.75)
**Patient Satisfaction questionnaire**	1 (0.75)
	
**Type of SOMs per domain (n=133)**	
***Domain 1: Impairment-based tools (n=81)***	
**Pain Numerical Rating Scale**	26 (32.1)
**Goniometer**	16 (19.8)
**Oxford Scale/Manual Muscle Testing**	11 (13.6)
Others*	28 (34.6)

***Domain 2: Function/participation-oriented tools (n=50)***	
**Oswestry Disability Index**	19 (38.0)
**Lower Extremity Functional Scale**	12 (24.0)
**Quick DASH**	9 (18.0)
Others**	10 (20.0)

***Domain 3: Health-related quality of life tool (n=1)***	
**SF-36**	1 (100)

***Domain 4: Patient satisfaction SOMs (n=1)***	
**Patient Satisfaction Questionnaire**	1 (100)

**Reasons facilitating SOMs utilisation (n=47)**	
**Availability of the SOMs**	33 (70.2)
**Inexpensive to acquire**	27 (57.4)
**Easy methodological procedures to follow**	23 (48.9)
**Less time consuming to use**	19 (40.4)
**Does not require a lot of human resources**	15 (31.9)
**Logical acceptability/relevance of the SOMs**	14 (29.8)
**Ease of scoring and interpretation the scores**	10 (21.3)
**Ensures patient safety**	9 (19.1)
**Others**	11(23.4)

SF-36=short form 36; DASH=Disabilities of the Arm, Shoulder and Hand; Paper-based outcome measures are tools questionnaire related tools; Function/performance-based outcome measures are tools measuring function or performances; Technical/instrument-based outcome measures are outcomes assessed using instruments requiring the use of PT technique such as goniometry; Clinically-administered tests are clinical tests performed by PTs during clinical examination procedures

* refers to other types of impairment based tools

** refers to the other types of function or participation-oriented tools

In total, 133 distinct SOMs were identified as being used by PTs during the specified period. Based on the reports of the 47 participants routinely using SOMs, the clinically administered tests were popular (n=47, 100%) followed by paper-based outcome measures (n=39, 83.0%). Further classification of the 133 tools by domain showed that most SOMs utilised by PTs in clinical practice in the last six months were mainly impairment-based tools (n=81, 60.9%). Variably, the tools were assessing pain intensity, joint range of motion, muscle strength, muscle flexibility, muscle tone, dynamic and static balance, joint proprioception, soft tissue oedema, and respiratory difficulties, among others. Specifically, the Pain Numerical Rating Scale (PNRS), Goniometry and the Oxford Scale were consistently used as impairment-based SOMs. Among the SOMs evaluating functional limitations and participation restrictions, the Oswestry Disability Index (ODI), Lower Extremity Functional Scale (LEFS), and Quick Disability of the Arm, Shoulder and Hand (Quick DASH) were the most commonly used.

The 36-Item Short Form Survey (SF-36) and Patient Satisfaction Questionnaire Short Form (PSQ-18) were the only SOMs highlighted for evaluating HRQoL and patient satisfaction with physiotherapy treatment, respectively. Thematic analysis of the perceived reasons for frequently utilising the above-mentioned SOMs showed that PTs had multifarious reasons. The most common reasons highlighted were broadly categorised into (i) easy availability of the SOMs; (ii) cost-related concerns; (iii) methodological/procedural considerations, (iv) time-related factors; (v) resources; (vi) logical acceptability or relevance of the SOMs; (vii) ease of scoring and interpretation of the scores; (viii) and patient safety concerns with regards to the SOMs administration.

### Perceived benefits and barriers of using SOMs

Table 3 depicts the benefits of using SOMs as perceived by all the participants regardless of utilisation status. The majority of the participants (n=75, 79.8%) reported that SOMs helps with accountability and objectivity during patient assessment. Moreover, 76.0% (n=73) strongly agreed that SOMs direct plan of care for patients.

**Table 3:** Perceived benefits of using outcome measures among physiotherapists (n=99)*

Perceived benefits of SOMs	Total responses** N	Strongly Agree n(%)	Agree Somewhat n(%)	Completely Disagree n(%)
**Help direct the plan of care**	96	73 (76.0)	18 (18.8%)	5 (5.20)
**Enhance communication between PTs and patients**	94	55 (58.5)	33 (35.1)	6 (6.38)
**Enhance communication with third-party payers, doctors and other providers such as medical aid organisations**	93	55 (59.1)	31 (33.3)	7 (7.52)
**Help patients feel that PTs are thorough in their examination.**	94	53 (56.4)	33 (35.1)	8 (8.51)
**Helps with patient satisfaction related aspects**	94	46 (48.9)	41 (43.6)	7 (7.44)
**Increases the efficiency of clinical examinations.**	94	63 (67.0)	20 (21.3)	11 (11.7)
**Help focus choice of physiotherapy interventions.**	93	60 (64.5)	25 (26.9)	8 (8.60)
**Attaining better patient outcomes.**	94	56 (59.6)	33 (35.1)	5 (5.31)
**Helps to motivate and encourage patients**	94	53 (56.4)	31 (32.9)	10 (10.7)
**Help with compliance and adherence to hospital/clinic visits**	93	32 (34.4)	47 (50.5)	14 (15.1)
**Decreases the rates of denial from third-party payers**	93	34 (36.6)	44 (47.3)	15 (16.1)
**Enhanced marketing of my practice or services.**	94	40 (42.6)	44 (46.8)	10 (10.6)
**Helps with accountability and objectivity with assessment and treatment**	94	75 (79.8)	15 (16.0)	4 (4.25)

* represents the total number of participants requested to provide information on the perceived benefits of using SOMs among PTs and interns

** represents the total number of responses for each question related to the perceived benefit

All the participants were requested to provide information on possible barriers to SOMs utilisation. About a quarter of the participants (n=41, 43.6%) strongly agreed that they are deterred from using SOMs because of the language of construction of the tool (Table 4). They felt that most SOMs are in English, a language perceived to be difficult for patients to comprehend. A sizeable proportion (36.2%, n=34) also perceived that many SOMs require a higher reading proficiency level that is disproportionate to the patient population.

**Table 4:** Perceived barriers to utilisation of standardised outcome measures

Perceived barriers	Total responsesn	Strongly Agree n (%)	Agree Somewhat n (%)	Completely Disagree n (%)
**They are confusing to patients**	94	7 (7.44)	40 (42.6)	47 (50.0)
**They are difficult for patients to complete independently**	93	**32 (34.4)**	43 (46.2)	18 (19.4)
**They require too high a reading level for many patients**	94	**34 (36.2)**	37 (39.4)	23 (24.5)
**Most are in English, a language in which many of my patients are not fluent.**	94	**41 (43.6)**	33 (35.1)	20 (21.2)
**They are not sensitive to the cultural/ethnic concerns of many patients**	93	19 (20.4)	42 (45.2)	32 (34.4)
**They make patients anxious.**	92	16 (17.4)	39 (42.4)	37 (40.2)
**They take too much time for patients to complete.**	94	**32 (34.0)**	43 (45.7)	19 (20.2)
**They take too much time for PTs to calculate/score/analyse**	94	16 (17.0)	55 (58.5)	23 (24.5)
**They provide information that is too subjective to be useful.**	94	9 (9.57)	35 (37.2)	50 (53.2)
**They require more effort than they are worth**	94	10 (10.6)	28(29.8)	56 (59.6)
**They do not contain information that helps me direct the plan of care.**	94	9 (9.57)	23(24.5)	62 (66.0)
**They are difficult to interpret (e.g., do not know what norms are, how score relates to severity, or what a clinically important change might be).**	94	14 (14.9)	40 (42.6)	40 (42.6)
**They do not contain the types of items/questions relevant for the type of patients I see.**	93	11(11.8)	26 (28.0)	56 (60.2)
**Often do not get completed at discharge, so cannot give information about patients' response to treatment.**	93	**28 (30.1)**	47 (50.5)	18 (19.4)
**There are many different SOMS measuring one construct, I struggle choosing which one to use**	96	**30 (31.3)**	39 (40.6)	27 (28.1)

Bolded represents the most common barriers.

## Discussion

The present study provides evidence on perceived facilitators and barriers to SOMs utilisation by PTs in Namibia. Although there is substantial evidence in the global literature, the Namibian context, disproportionately composed of private practitioners and PTs with diverse educational backgrounds, provides a distinct perspective on the available evidence. The present study showed that PTs in Namibia used multiple SOMs in clinical practice and recognised the benefits of routinely collecting outcome measures. Given the heterogeneous nature of the sample with respect to educational background and clinical experience, these findings are interesting and provide vital contextual evidence on the level of awareness of Namibian PTs with regard to the importance of utilising SOMs. This heightened awareness may be linked to the participants' educational or professional training on the use of SOMs.

The reported high level of awareness of the benefits of SOMs only translated to half of the PTs routinely utilising the SOMs. Although consistent with findings in the literature showing low to moderate utilisation rates in many African clinical settings, the present study's findings are concerning, as they do not reflect the ideal standard of practice advocated by regulatory bodies such as WP.^7^,^9^ With the advent of evidence-based practice and burgeoning pressure to measure the impact of interventions, PTs are challenged to use SOMS every time.^2^ However, this has remained aspirational in Namibia with the present study providing evidence of ambivalence towards outcome measurement by PTs. Cognisant of the challenges hindering utilisation, efforts to formulate policies and strategies to improve utilisation should be prioritised.

Local regulatory councils and professional associations, such as the Namibia Society of Physiotherapists (NSP), should collectively develop clinical practice guidelines emphasising, among other things, routine utilisation of SOMs and further superintend the implementation of the guidelines. Currently, there are no formulated clinical practice guidelines clearly mandating and reinforcing the use of SOMs in Namibia. This is a significant shortcoming with regard to the monitoring and evaluation of SOMs utilisation in the country.

In the present study, more than one hundred different SOMs were listed as commonly used in clinical practice for the specified period. This figure contrasted the 51 different SOMs reported by Sawadogo et al which recruited 241 PTs from 13 different Sub-Saharan African countries.^15^ Although the present study findings are remarkable in highlighting the significant diversity in the type of SOMs utilised by PTs in Namibia, the combined results illustrate the influence of context in determining the specific type of SOMs used by PTs worldwide. Collectively, the findings illuminate the relevance of considering contextual determinants in ensuring successful routine outcome measurement. Plausibly, the diversity in SOMs utilised in the Namibian context is explained by the range of symptoms managed by PTs.

This study showed that PTs treated a wide range of patient symptoms including pain, joint motion restrictions, muscle performance dysfunctions, gait disturbances, swelling, inefficient functional movement patterns, breathing problems, impaired balance, postural control and stability.

Further profiling of the commonly used SOMs in the current study revealed that participants preponderantly utilised impairment-oriented SOMs. There is limited incorporation of SOMs evaluating changes in functional limitations, participation restrictions, HRQoL and patient satisfaction in Namibia. This disagrees with the WP drive emphasising the routine evaluation of functional status and HRQoL as key health outcomes to check effectiveness of PT interventions.^6^ Contextually, these findings add support to the urgent need of developing consensus-derived clinical practice guidelines stipulating the categories of SOMS to be frequently used in accordance with the profession scope of practice.

Although the specific reasons explaining why PTs favour impairment-based SOMs are unclear from the current study, innumerable factors possibly account for that surprising finding. Firstly, the fact that all PTs reported utilising a variety of clinically-administrable special tests during patient physical examination procedures could be an important explanatory and contributing finding. Expectedly, PTs use special tests as part of standard physical examination procedures either for diagnostic purposes or baseline evaluation of impairments affecting function. Hence, tests such as Manual Muscle Testing, for example, are likely to be popular among PTs. Invariably, PTs' physical examination procedures entail assessment of clinical variables such as tenderness, active and passive joint range of motion, muscle strength, muscle endurance, muscle tone, muscle flexibility, balance, and proprioception, which necessitate the use of clinically-administrable tests.

Secondly, since most impairment-oriented SOMs are either clinically administrable or technical/instrument-based tests, they render them convenient to use as opposed to paper-based SOMs. This possibly explains some of the perceived facilitators and barriers highlighted by the sample participants in the present study. Participants were inclined to administer SOMs that were inexpensive, easily available, easy to use and interpret the scores, did not require too many resources in terms of time and personnel and were logically acceptable to them. These findings on facilitators have also been shared in previous studies.^13^

Broadly, the main barriers for SOMs highlighted by the PTs can be categorised into patient, PT and SOMs-related factors. These findings agree with published evidence.^7^,^9^,^13^,^16^ The participants in the present study felt that most SOMs were in English, a language difficult for most patients to understand creating challenges with independent tool completion.

These findings expose a major problem that could perpetuate depressed utilisation rates in Namibia unless drastic measures are taken.

This problem particularly affects the administration of paper-based SOMs. Besides exhorting the PTs to administer the SOMs using an interview-based strategy instead of patients independently completing the tool, there is need to augment scientific evidence on the most commonly-used outcome measures in Namibia by conducting repeat studies with robust study designs such as Delphi studies. Subsequently, the identified tools could then be construct-validated locally, translated into local languages and stipulated in the policy clinical guidelines as SOMs of choice. The current study showed that the ODI, LEFS and Quick DASH questionnaires were popular among Namibian PTs. That finding offers a critical starting point for cross-cultural validation of the identified tools in Namibia. Another barrier of importance highlighted by participants was that of time; a finding also shared in literature.^9^,^15^

The participants felt that most SOMs require ample time to complete. This is particularly evident for performance or paper-based SOMs assessing function and HRQoL. For example, SF-36 was named in this study for the assessment of HRQoL. This validated tool has 36 questions which have to be completed for correct summative evaluation of patients HRQoL status. Patients may struggle completing the tool independently and given the busy nature of the PTs clinics/hospital departments, treating many patients per day, the probability of long SOMs being used is negligible. However, the time barrier requires a mental shift in the attitude of individual PTs impacted through continuous professional development and stringent local regulatory policies to register any improvement in the utilisation rates of paper-based SOMs which appear long or time-consuming to complete.

### Critical assessment of the study

This study profiled the utilisation of SOMs by PTs in Namibia with the aim of documenting evidence on perceived facilitators and barriers. The study recruited a sample of PTs derived from both private and public sector, working in all administrative regions of Namibia providing external validity to the results and negating non-participation bias. Furthermore, instrument questions were adopted from related previous studies making our results potentially comparable to others. Furthermore, the adapted tool was subjected to logical validity and test-retest reliability assessment yielding satisfactory indices. Nonetheless, the study had limitations.

The research approach used was a cross-sectional study to identify barriers, which cannot be alluded to as the actual causes of reduced utilisation rates in Namibia. Further repeat studies with robust study designs are still needed to explore the phenomenon of SOMs utilisation. The study relied on PTs' self-reports based on experience in clinical practice for the last six months prior to data collection. This creates a possibility of recollection bias, which can over-or underestimate the utilisation rates and further influence the recalling of either the perceived barriers the PTs experienced hindering SOMs utilisation or actual facilitators which promoted the uptake and implementation of SOMs during the specified period.

## Conclusion

The present study showed that about half of the participants routinely used different SOMs, particularly impairment-based. Consequently, there is a need to raise awareness through different platforms to encourage utilisation of SOMs that encompass the assessment of function, participation and HRQoL. This will ensure that PTs are holistic in their clinical approach and abreast with empirical evidence.

The most common reasons facilitating SOMs usage included tool availability and affordability. However, most participants perceived that time constraints and tool-related factors, such as language and the complexities of the questions in the tool, hindered the routine administration of the SOMs. These findings warrant local regulatory councils and professional associations to collectively develop clinical practice guidelines emphasising routine utilisation of SOMs and superintend the implementation of the guidelines by registered PTs in Namibia. However, there is a need for the adoption of contextually validated and translated tools, particularly for the paper-based SOMs, which can be stipulated as the SOMs of choice.
